# Simultaneous green TLC determination of nirmatrelvir and ritonavir in the pharmaceutical dosage form and spiked human plasma

**DOI:** 10.1038/s41598-023-32904-x

**Published:** 2023-04-15

**Authors:** Mohamed S. Imam, Ahmed H. Abdelazim, Afnan S. Batubara, Mohammed Gamal, Ahmed A. Almrasy, Sherif Ramzy, Hanan Khojah, Tamer H. A. Hasanin

**Affiliations:** 1 Pharmacy Practice Department, College of Pharmacy, Shaqra University, Shaqra, 11961, Saudi Arabia; 2 Clinical Pharmacy Department, National Cancer Institute, Cairo University, Fom El Khalig Square, Kasr Al-Aini Street, Cairo, 11796 Egypt; 3 Pharmaceutical Analytical Chemistry Department, Faculty of Pharmacy, Al-Azhar University, Nasr City, 11751 Cairo Egypt; 4 Department of Pharmaceutical Chemistry, College of Pharmacy, Umm Al-Qura University, Makkah, Saudi Arabia; 5 Pharmaceutical Analytical Chemistry Department, Faculty of Pharmacy, Beni-Suef University, Beni-Suef, 62514 Egypt; 6 Department of Phramacognosy, Faculty of Pharmacy, Jouf University, Sakaka, Aljouf Saudi Arabia; 7 Department of Chemistry, College of Science, Jouf University, Sakaka, Aljouf Saudi Arabia; 8 Department of Chemistry, Faculty of Science, Minia University, El-Minia, 61519 Egypt

**Keywords:** Drug safety, Drug screening, Chemistry, Analytical chemistry, Green chemistry

## Abstract

Quantitative analysis of pharmaceutical compounds up to Nano gram levels is highly recommended to introduce feasible and sensitive tool for determination of the compounds in the pharmaceutical and biological samples. Nirmatrelvir plus ritonavir was recently approved in the US, the UK and Europe as a new co-packaged dosage form for the treatment of COVID-19. The objective of this work was to develop a more sensitive TLC method based on using β-cyclodextrin as a chiral selector additive in the mobile phase for simultaneous determination of nirmatrelvir and ritonavir in pure form, pharmaceutical formulation and spiked human plasma. The analysis procedures were developed using TLC aluminum silica gel plates and methanol–water- 2% urea solution of β-cyclodextrin (40:10:.5, by volume) as a mobile phase with UV detection at 215 nm. The developed method was successfully applied over a linearity range of 10–50 ng/band for both nirmatrelvir and ritonavir. The method was validated for limits of detection and quantitation, accuracy, precision, specificity, system suitability, and robustness. Furthermore, the eco-friendliness of the proposed method was assessed using the analytical eco-scale and the green analytical procedure index. The described method exhibited compliance with green analytical chemistry principles based on common green metric values.

## Introduction

In quantitative pharmaceutical analysis, improving the sensitivity and selectivity of analytical procedures is a key objective for researchers. It is highly recommended to determine compounds of interest at Nano gram levels to provide a feasible tool for assaying compounds in a wide range of matrices. Thin-layer chromatography (TLC) is a commonly used separation technique for pharmaceutical quantitative analysis of binary or multicomponent mixtures, owing to the affordable cost of the equipment and the ease of laboratory preparation of the stationary phase plates^1–3^.

Cyclodextrins are oligosaccharides composed of six, seven, or eight glucose units (α, β, or γ units). They are toroidal in shape, with a hydrophobic inner cavity and hydrophilic external surface. Cyclodextrins enable the selective binding of various organic, inorganic, and biological guest molecules into their cavities and form stable host–guest inclusion complexes^4,5^. The selective association of different molecules with the hydrophobic cavities of cyclodextrins enhances the targeting goals of analysis procedures, depending on the strong recognition and enrichment functions of the cyclodextrins^6^. Previous reports demonstrate the role of stationary phase modifiers and mobile phase additives in improving the efficiency of TLC procedures^7–10^. β-cyclodextrin [β-CD] is one of the chiral selectors that can be added to the mobile or stationary phase to improve the resolution of different compounds^11,12^.

**Figure 1:**
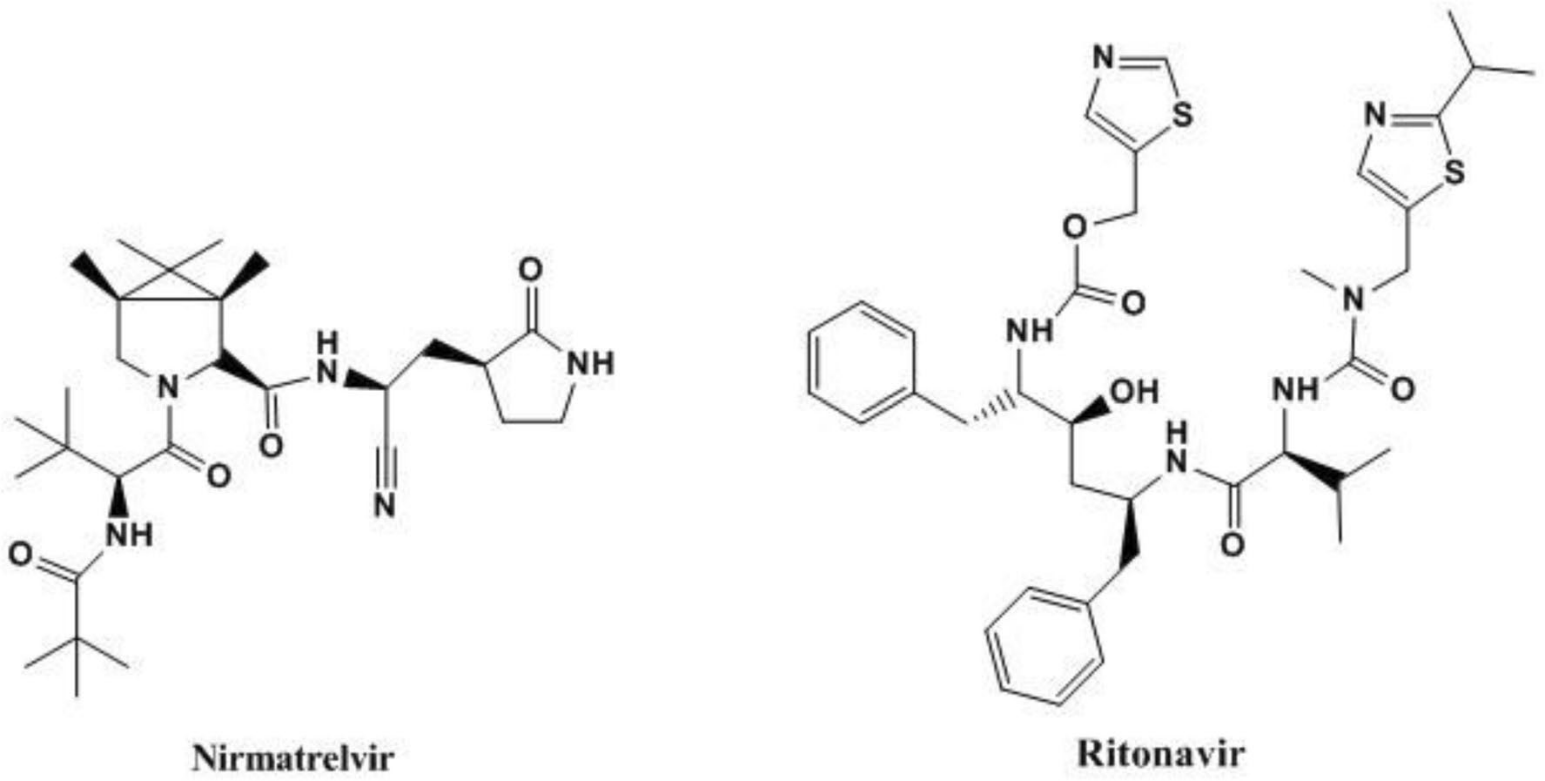
Structural formula of nirmatrelvir and ritonavir.

Nirmatrelvir, Fig. 1, is a peptidomimetic inhibitor of the major protease of severe acute respiratory syndrome coronavirus 2, while ritonavir, Fig. 1, is a protease inhibitor of human immunodeficiency virus type 1 and also a CYP3A inhibitor. Nirmatrelvir plus ritonavir was recently approved in US, United Kingdom and Europe as a new co-packaged dosage form for the treatment of COVID-19 in adults who are at high risk of infection^13–17^.

LC–MS/MS was previously developed for determination of nirmatrelvir and ritonavir in the human plasma sample^18^. To date, no analytical methods have been introduced for determination of the drugs under the study in the new FDA approved co-packaged dosage form. The objective of this work was to develop highly selective and sensitive TLC procedures based on using β-CD chiral selector as mobile phase additive for simultaneous determination of nirmatrelvir and ritonavir in the pure, pharmaceutical and spiked human plasma. The analysis procedures were developed using TLC aluminum silica gel plates, methanol–water- 2% urea solution of β-CD (40:10:0.5, by volume) as a mobile phase and UV detection at 215 nm. The proposed TLC densitometry method was efficient for the estimation of nirmatrelvir and ritonavir up to Nano gram levels. Furthermore, the green compliance of the described TLC method was measured using the analytical eco-scale and the green analytical procedure index. The described method exhibited an agreement with the green analytical chemistry principles in terms of the common green metric values.

## Experimental

### Materials

The reference standard samples of nirmatrelvir (99.36%), ritonavir (99.62%) and paxlovid tablets [150 mg nirmatrelvir tablets co-packaged with 100 mg ritonavir tablets] and prescribed in the ratio 2.1, were kindly supplied by Pfizer, Inc., Egypt.

### Chemicals and solvents

Methanol, HPLC grade (Sigma-Aldrich, Germany). β-CD, 98%, (Sigma-Aldrich, Germany). Aqueous solution of β-CD was prepared as, 2% w/v in 30% w/v aqueous urea solution.

### Apparatus

Camag TLC scanner 3, with WINCATS computer software (Switzerland). TLC plates, silica gel 60 GF_254_ (20 × 20 cm), (Fluka chemie, Switzerland). Hamilton 50-µL microsyringe (Germany). Chromatographic tank (25 × 25 × 9 cm).

### Standard solutions

Standard solutions of nirmatrelvir and ritonavir (100 μg/mL) were prepared by dissolving 10 mg of each drug powder in 50 mL of methanol using a 100-mL volumetric flask and completing to volume with methanol.

### General procedures

#### TLC densitometry conditions

TLC Densitometry separation process was developed through auto sampling of the prepared solutions on pre-coated TLC aluminum silica gel 60 GF254 plates (20 × 20 cm). 10 μL of sample were spotted 8 mm from the edge of the plates. The plates were developed to a distance of 85 mm in a chamber previously saturated with the mobile phase methanol–water- 2% urea solution of β-CD (40:10:5, by volume) for 30 min. The densitometry measurements were conducted in reflectance absorbance mode at wavelength of 215 nm. The scanning speed was 20 nm/s and the data resolution was 100 μm/step. The slit dimension was kept at 6.0 × 0.3 µm. The described analysis process was repeated three times and the results were determined.

#### Construction of calibration graphs

Aliquots of a standard solution nirmatrelvir and ritonavir solution (100 μg/mL) corresponding to (10–50 μg) of each drug were transferred to a series of 10-mL volumetric flasks and diluted to volume with methanol. 10 μL of the prepared solutions were applied and spotted on the TLC plate under the specific TLC densitometry conditions. The average values of band areas of nirmatrelvir and ritonavir were plotted against the drug concentrations (ng/band) to construct the calibration graphs. Regression data were obtained. In general, all described procedures were performed in accordance with relevant guidelines.

#### Laboratory prepared mixture analysis

Different laboratory mixtures of nirmatrelvir and ritonavir in various ratios, considering the prescribed assigned pharmaceutical dosage administration (nirmatrelvir, ritonavir 2:1), were done by diluting the mixtures with methanol. These laboratory mixtures were then analyzed using the described procedures and the corresponding concentration of nirmatrelvir and ritonavir were calculated from the regression equation.

### Procedures for co-packaged pharmaceutical tablets

Nirmatrelvir pharmaceutical tablets (2 units) and ritonavir pharmaceutical tablet (1 unit) were crushed, weighed, and powdered. The accurately weighted powders were added to a 100-mL volumetric flask and made up to 50 mL with methanol. The resulting solution was shaken and sonicated for 20 min, and then filtered. The final volume was adjusted to 100 mL with methanol to prepare a solution containing 3 mg/mL nirmatrelvir and 1 mg/mL ritonavir. Different working solutions were prepared with methanol to obtain various concentrations of nirmatrelvir and ritonavir. These solutions were analyzed using the TLC procedure as prescribed and the corresponding concentrations were calculated from the regression equation.

### Procedure for spiked human plasma

Different spiked human plasma samples of nirmatrelvir and ritonavir were prepared by transferring aliquots of the mentioned drugs at different concentrations to a series of 10-mL centrifugation tubes, along with 1 mL of human plasma and 3 mL of acetonitrile. The tubes were shaken for 1 min on a vortex mixer and then centrifuged for 30 min. The resulting supernatants were evaporated to dryness and the residues were dissolved in appropriate volume of methanol. They were then transferred to 10-mL volumetric flasks, along with 3 mL of acetate buffer pH 4, and diluting to volume with methanol. The samples were analyzed using the TLC procedure as previously described, and the corresponding concentrations were calculated from the regression equation.

### Ethical approval and consent to participate

This work was approved by the Committee of Research Ethics in the Cairo Faculty of Pharmacy, Al-Azhar University, Egypt.

## Results and discussion

Development of quantitative analytical methods with higher sensitive detection limits and targeting selectivity of compounds of interest represents attractive approach for researchers. The ability to quantitatively analyze drugs at extremely low levels, in the Nano gram range, allows for their detection in biological fluids. One such drug combination is nirmatrelvir and ritonavir, which is a recently approved co-packaged medication by the FDA for the treatment of COVID-19^13–17^. As these drugs have recently been introduced to the market, it is advisable to establish a validated analytical method for detecting nirmatrelvir and ritonavir in their pure form, as well as in their pharmaceutical form and in spiked plasma samples.

In the recent work, the strategy was to develop higher sensitive selective TLC procedures, using β-CD chiral selector as mobile phase additive, for efficient simultaneous determination of nirmatrelvir and ritonavir at the Nano gram levels. Furthermore, the green characters of the described method were tested using the analytical eco-scale and the green analytical procedure index. The results revealed the fitness of the proposed method to the green analytical chemistry metric values.

### Optimization of TLC densitometry procedures regarding to selectivity and sensitivity goals

The efficiency of the TLC process depends mainly on the nature of the stationary phase, the chemical properties of the compounds intended to be separated and the elution strength of the mobile phase.

### TLC stationary phase characters and the proposed interaction with nirmatrelvir and ritonavir

Silica gel is the common stationary phase in the TLC procedures, and it is composed of a network of silicon-oxygen bonds and (Si–OH) groups on the surface. TLC separation process occurs due to the variations in the migration of sample molecules caused by selective hydrogen bonding and electrostatic interactions with the silica surface. The strength of intermolecular forces between the sample, the stationary phase (which has high polar characters), and the organic mobile phase (which is the mostly less polar) affects the equilibrium distribution between the two phases. More polar compounds tend to attract with the stationary phase rather than the less polar mobile phase, resulting in lower R_f_ value and vice versa^19,20^. The structural formulas of nirmatrelvir and ritonavir vary in terms of polar characters. Ritonavir is a highly hydrophobic, non-polar compound which is predicted to spend less time in the stationary phase and more time in the organic mobile phase. Ritonavir traveled the furthest up the plate and had the highest R_f_ value of 0.41. On the other hand, nirmatrelvir is relatively hydrophilic with polar characters allowing it to hydrogen bond to the polar stationary phase and reflect a strong attraction to the stationary phase compared to ritonavir. Nirmatrelvir spent more time in the stationary phase and less time in the mobile phase resulting in lower R_f_ value of 0.14.

### Mobile phase selection and the proposed interaction with nirmatrelvir and ritonavir

Proper selection of solvent is essential for an efficient TLC procedure, and determining the best solvent often requires multiple trials and errors. One option for improving the resolution of different compounds is to add β-CD, a chiral selector, to either the mobile or stationary phase. The inclusive complex formed between β-CD and different compounds occurs through the asymmetric centers placed within β-CD, which interact via hydrogen bonds with corresponding groups of the compounds being studied. The toroidal shape of β-CD with a hydrophobic inner cavity and hydrophilic edge, enables efficient interaction with molecules bearing groups of various polarities. β-CD also plays an essential role as a mobile phase additive for selective TLC separation processes^4–7^. Although the use of β-CD in TLC was limited due to solubility difficulties in water, it can be dissolved in aqueous urea solution which making it suitable for the TLC separation processes.

Solvent systems with different ratios of methanol–water and methanol–water-β-CD were tested. The mobile phase of methanol–water gave closely bands of nirmatrelvir and ritonavir with poor resolution and less symmetric pattern. In order to enhance the resolution, small volume of β-CD was added to the mobile phase. The best separation with well-defined bands was obtained after the mobile phase became methanol–water- 2% urea solution of β-CD (40:10: 5, by volume) and after the chamber was saturated with the mobile phase for 20 min at room temperature. The selected mobile phase allowed the simultaneous determination of nirmatrelvir and ritonavir without tailing of the separated bands.

In addition, theoretical examination of the binding between nirmatrelvir and ritonavir with β-CD was done. Gauss-view software was used to create and optimize nirmatrelvir, ritonavir, β-CD, and their corresponding complex products. The optimized products' energy values were evaluated using the density functional theory method with the B3LYP/6-31G (d) basis set level. To determine the binding energy of nirmatrelvir and ritonavir with β-CD, the following equation was employed^21^:

$$\Delta {\text{E}} = {\text{E}}_{{{\text{A}}{-}{\text{B}}}} - {\text{E}}_{{\text{A}}} {-}{\text{E}}_{{\text{B}}}$$

where A is the energy of the molecular structure of the nirmatrelvir or ritonavir, B is the energy of the molecular structure of β-CD and ∆E is the binding energy.

The calculated binding energy for complexes formed between ritonavir and nirmatrelvir with β-CD was − 166.7 kJ/mol and − 122.5 kJ/mol, respectively. The differences in the calculated binding energy between the above drugs and β-CD indicate that ritonavir binds more strongly to β-CD than nirmatrelvir which assured the obtained experimental result. To assess the potential impact of solvents on the stability and energy of optimized products in the presence of urea versus the gaseous phase, the polarizable continuum model^22^ was used to calculate the energy values of the optimized products. This model considers the effects of solvents during energy calculations. In the presence of urea, the binding energy is quite similar to that obtained in gaseous phase, which suggests that binding is strong enough to persist also in solution phase.

### Wavelength selection

**Figure 2:**
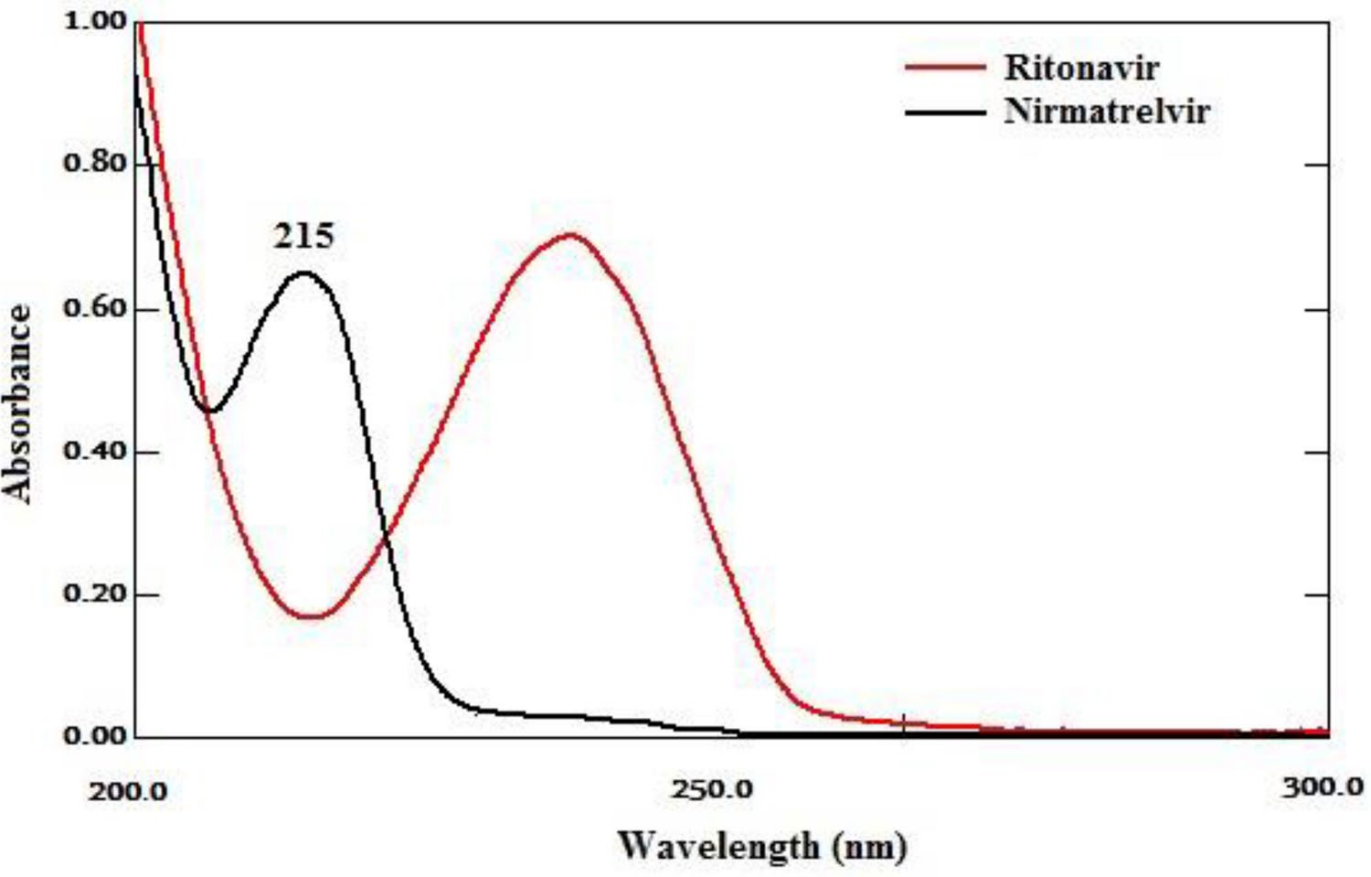
UV spectra of nirmatrelvir (15 μg/mL) and ritonavir (15 μg/mL).

After checking the UV spectra of nirmatrelvir and ritonavir, Fig. 2, together with testing different wavelengths, 215, 225 and 235 nm. A wavelength of 215 nm was chosen since it provided a good sensitivity with sharp detection for the drugs under the study (Table 1).

**Table 1:** Regression and validation results obtained for determination of nirmatrelvir and ritonavir by the described TLC densitometry method.

Parameters	Nirmatrelvir	Ritonavir
Scanning wavelength (nm)	215	215
Linearity range (ng/band)	10–50	10–50
Slope	478.27	974.66
Intercept	386.52	133.29
Coefficient of determination (r^2^)	0.9996	0.9999
LOD (ng/band)	0.695	0.430
LOQ (ng/band)	2.106	1.304
Accuracy (%R)^a^	100.38	98.75
Precision, (%RSD)^a^		
Repeatability	0.528	0.721
Intermediate precision	0.870	0.882
Robustness, (% R ± %RSD)		
Detection wavelength	99.87 ± 0.681	99.36 ± 0.369
Saturation time	100.49 ± 0.982	98.54 ± 0.597

^a^Values calculated for 9 samples (3 concentrations repeated 3 times).

### TLC system suitability parameters

To confirm that the TLC densitometry system was properly worked during the analysis operation, parameters including resolution factor (Rs), retention factor (k^−^), and tailing factor (T) were measured^23^. The results, Table 2, revealed that the developed TLC procedures provided complete baseline separation of nirmatrelvir and ritonavir bands with minimal tailing.

**Table 2:** TLC system suitability testing parameters for the determination of nirmatrelvir and ritonavir.

Parameters	Obtained value		Reference value^23^
	Nirmatrelvir	Ritonavir	
Retardation factor (R_f_)	0.14	0.41	–
Retention factor (K′)	9.00	1.70	1–10
Tailing factor (T)	1.50	1.10	> 2
Resolution (Rs)	4.13		< 2

**Figure 3:**
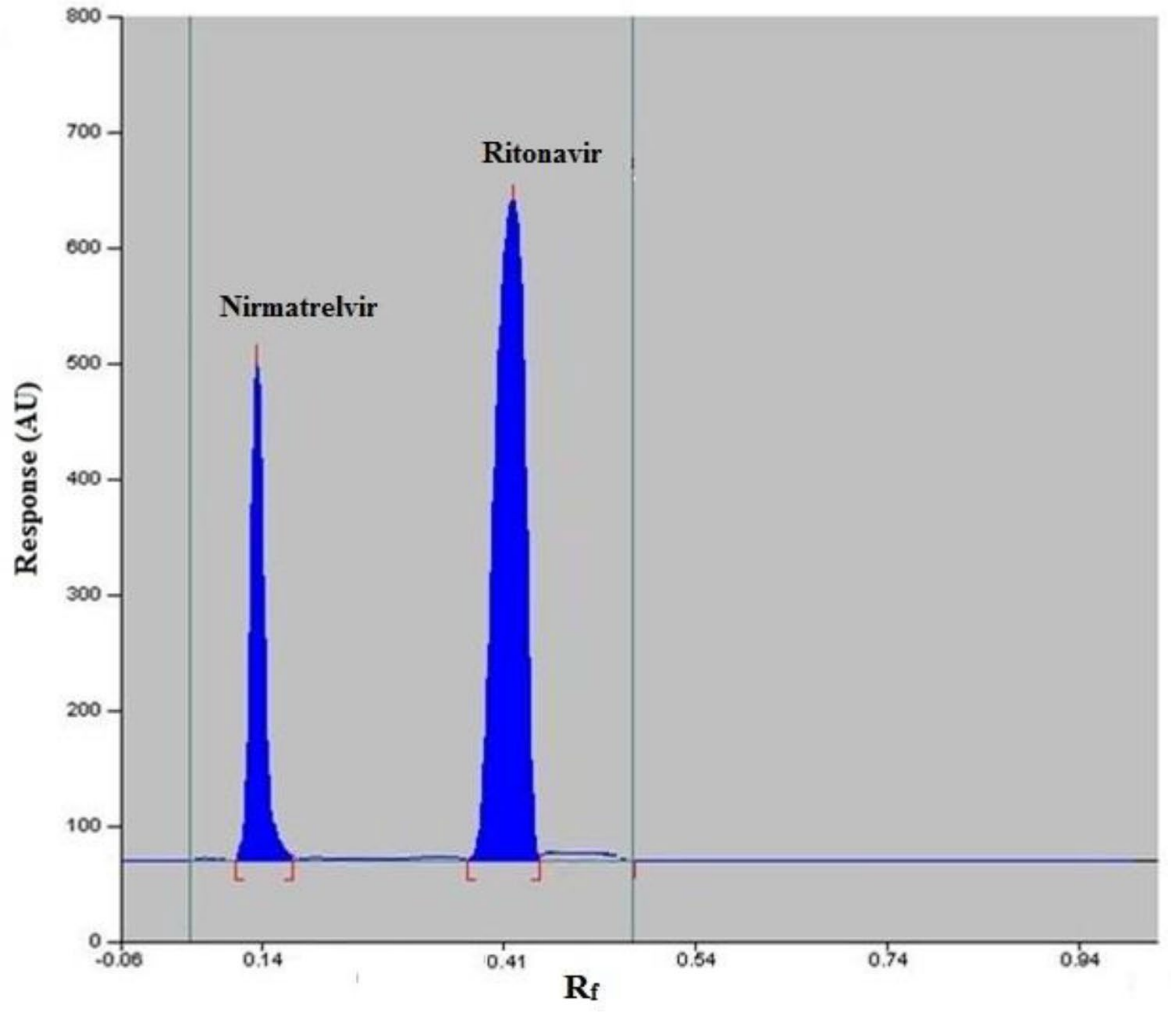
TLC densitogram of nirmatrelvir (15 ng/band) and ritonavir (15 ng/band).

After optimization of the TLC densitometry conditions, and visualization of the plates at 215 nm, TLC bands appeared at R_f_ of 0.14 for nirmatrelvir and 0.41 for ritonavir as shown in Fig. 3.

### Method validation

The method was tested for linearity, limits of detection (LOD) and limits of quantitaion (LOQ), acuracy, precision, specificity, system suitability and robustness. The linearity of the described method was tested and the calibration graphs were created by plotting the obtained band area values of the drugs under the study versus the corresponding concentrations in ng/band. The regression data obtained, Table 1, indicated that linearity was found over the range of 10–50 ng/band for nirmatrelvir and ritonavir. The high values of coefficient of determination as well as the small values of slope and intercept revealed the accepted regression evaluated data.

According to ICH recommendations, LOD and LOQ were calculated based on the relative standard deviation of the regression line (SD) and the slope as the following equations:

$${\text{LOD}} = {3}.{3}\;{\text{SD}}/{\text{slope}}\quad {\text{LOQ}} = {1}0\;{\text{SD/slope}}$$

The obtained data listed in Table 1 indicated the sensitivity of the described method. Method accuracy demonstrates the closeness agreement between the value obtained and the accepted reference value. It was calculated by assessing the mean percent recovery (%R), for triplicate determination of three concentration levels of each drug (15, 30, 45 ng/band). The obtained data listed in Table 1 indicated the accuracy of the proposed method.

Method precision demonstrates the closeness agreement between a series of measurements obtained from multiple sampling of the same homogeneous sample using the proposed procedures. It was calculated by assessing the percent of relative standard deviation (%RSD), for triplicate determination of three concentration levels of each drug (15, 30, 45 ng/band) within one day for repeatability and on three successive days for Inter mediate precision. The small values of %RSD demonstrated high precision of the proposed method as listed in Table 1.

Method robustness indicates its efficiency to remain unaffected by small but deliberate variations in the method parameters. It was assessed by calculating %RSD through repeating the TLC procedures under slight changes in the described conditions as detection wavelength (± 2 nm) and saturation time (± 2 min). The obtained values %RSD revealed method robustness, Table 1.

Method specificity was confirmed by perfect simultaneous determination nirmatrelvir and ritonavir. TLC densitograms revealed that nirmatrelvir and ritonavir were well clearly separated from each other. The cited procedure was selectively applied to the determination of nirmatrelvir and ritonavir in a laboratory prepared mixture considering the commercial dosage administration [nirmatrelvir, ritonavir 2:1]. The results obtained, Table 3, revealed the successful application of the described method for specific determination of the compounds of interest.

**Table 3:** Determination of nirmatrelvir and ritonavir in the laboratory prepared mixtures.

Nirmatrelvir added, (ng/band)	Ritonavir added, (ng/band)	Nirmatrelvir found, (ng/band)	Ritonavir found, (ng/band)	Nirmatrelvir, (% R)	Ritonavir, (% R)
30.00	10.00	29.84	9.87	99.46667	98.7
36.00	12.00	35.66	12.11	99.05556	100.9167
45.00	15.00	45.88	14.95	101.9556	99.66667
Mean ± %RSD				100.16 ± 1.57	99.76 ± 1.11

### Application of TLC method for simultaneous determination of nirmatrelvir and ritonavir in the pharmaceutical preparation and spiked human plasma

The described TLC procedure was used to determine nirmatrelvir and ritonavir in the pharmaceutical dosage form, Table 4. The obtained data were accepted and compared with the data obtained of the reported method^18^. Furthermore, the method was used for the determination of nirmatrelvir and ritonavir in the spiked human plasma as it provided a high degree of detection and allowed the determination of nirmatrelvir the mean plasma Cmax for ritonavir (0.84 µg/mL) and ritonavir, the mean plasma Cmax for ritonavir (2.21 µg/mL)^24^. The data obtained, Table 5, revealed that the described procedure is suitable for the determination of the drugs under the study in the spiked plasma without interference of endogenous components of the plasma matrix.

**Table 4:** Determination of nirmatrelvir and ritonavir in pharmaceutical tablets by the described and reported methods.

Parameters	Proposed method		Reported method^18^	
	Nirmatrelvir	Ritonavir	Nirmatrelvir	Ritonavir
Average (%Recovery)^a^	99.47	100.17	100.05	100.45
%RSD^a^	1.004	1.089	1.235	1.237
*t value* (2.306)^b^	0.370	0.370	–	–
*F value* (6.388)^b^	1.529	1.298	–	–

^a^Values calculated for five samples.

^b^The values in parenthesis are tabulated values of “*t*”and “*F*” at (*P* = 0.05).

**Table 5:** Determination of nirmatrelvir and ritonavir in the spiked human plasma samples.

Nirmatrelvir added, (ng/band)	Ritonavir added, (ng/band)	Nirmatrelvir found, (ng/band)	Ritonavir found, (ng/band)	Nirmatrelvir, (% R)	Ritonavir, (% R)
30.00	10.00	28.10	9.25	93.67	92.50
36.00	12.00	34.25	10.88	95.14	90.66
42.00	14.00	40.10	13.25	95.47	94.64
48.00	16.00	46.25	15.08	96.35	94.25
Mean ± %RSD				95.16 ± 1.12	93.01 ± 1.82

### Assessment of the green analytical chemistry fitness of the TLC method

**Figure 4:**
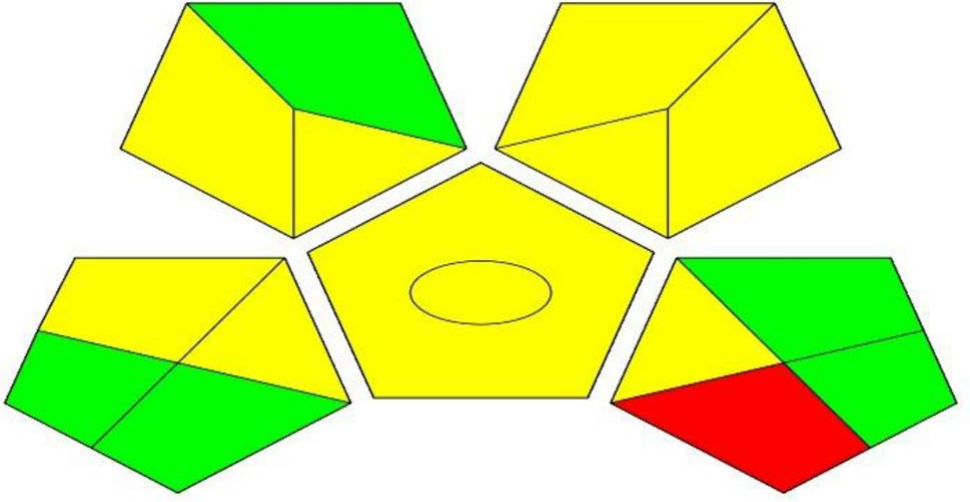
Green analytical procedure index pictogram symbol of the proposed TLC method.

Green fitness of the described TLC method was measured using, analytical eco-scale^25^ and green analytical procedure index^26^. The analytical eco-scale assessment tool evaluates the greenness level of the analytical method by calculating a numerical total score. An ideal green procedure would have a total score of 100 points with no penalty points. However, penalty points are subtracted from the total score for the harmful impacts the method may have on the environment, such as the consumption of hazardous solvents, high energy consumption, and the amount of waste generated. The assessment tool provides three classifications: the green method, which has a final total score of more than 75 points; the reasonably green method, which has a final total score between 50 and 75 points; and the inadequate green analysis, which has a final score below 50 points. Hazard penalty points are assigned based on the severity of the chemical's hazard, with non-hazard chemicals receiving no penalty points, less severe hazard chemicals receiving one penalty point, and more severe hazard chemicals receiving two penalty points^25^. The calculated score was 79, which indicated green matching of the TLC method with minimal negative impact on the environment. Additionally, a green analytical procedure index was introduced as a tool to evaluate the various steps involved in the proposed procedure. An Excel worksheet was used to measure the environmental impact of the analytical approach, utilizing a special symbol with pictogram parts that were color-coded green, yellow, and red, similar to traffic signals. Green indicated a safe procedure while red represented non-eco-friendly operations. The green analytical procedure index pictogram had five main sections, including sample preparation parameters, solvent and reagent parameters, instrumentation parameters, and an extra quantification mark^26,27^. Green analytical procedure index pictogram symbol of the proposed TLC method was shown in Fig. 4. The results demonstrated the environmentally friendly nature of the developed TLC method, as evidenced by the green assessment results**.**

## Conclusion

Higher sensitive validated TLC method was developed based on using β-cyclodextrin as a mobile phase additive, for simultaneous determination of nirmatrelvir and ritonavir in the pure form, pharmaceutical dosage and spiked human plasma. The developed method was successfully applied over the linearity range of 10–50 ng/band for nirmatrelvir and ritonavir. The major advantage of the described TLC densitometry method is that it can be performed with a small amount of solvent, which shortens the analysis time and enables cost-effective analytical procedures.

## Data Availability

The datasets used during the current study are available from the corresponding author on reasonable request.
